# Ultralow‐Energy Analog Reservoir Computing via Reconfigurable 2‐Transistor Memory

**DOI:** 10.1002/advs.77921

**Published:** 2026-09-29

**Authors:** Jeong‐Min Park, Yeong‐In Kim, Jeong Hyun Yoon, Sein Lee, Wooho Ham, Dongyun Lee, Junseo Lee, Dongwook Shin, Kyung Jun Park, Seok Daniel Namgung, Min‐Kyu Song, Jang‐Yeon Kwon

**Affiliations:** ^1^ School of Integrated Technology Yonsei University Incheon Republic of Korea; ^2^ BK21 Graduate Program in Intelligent Semiconductor Technology Yonsei University Incheon Republic of Korea; ^3^ School of Electrical Engineering Korea University Seongbuk‐gu Seoul Republic of Korea; ^4^ Department of Materials Science and Engineering Seoul National University Seoul Republic of Korea; ^5^ School of Electrical and Electronics Engineering Chung‐Ang University Seoul Republic of Korea

**Keywords:** 2‐transistor, InSnZnO, reconfigurable, reservoir computing, spatial compression

## Abstract

Reservoir computing (RC) offers a promising route to real‐time temporal information processing and is widely regarded as an energy‐efficient alternative to conventional von Neumann architectures. Paradoxically, many hardware RC systems have yet to demonstrate clear advantages in both array‐level energy efficiency and processing speed, despite these being among the field's central motivations. Furthermore, their practical viability is limited by structural complexity and insufficient robustness. Here, we report a reconfigurable, dynamic random‐access memory (DRAM)‐like physical RC platform based on an oxide channel two‐transistor (2T) cell, providing a practical route toward low‐energy, fast, robust, and structurally simple physical RC. Within a single device footprint and fabrication flow, the same 2T cell can be reconfigured to volatile reservoir dynamics or quasi‐non‐volatile readout memory. Its operating mode is selected by the hold bias, enabling electrical reconfiguration with tunable relaxation dynamics. This compact strategy enables spatiotemporal programmability while preserving process simplicity, as validated by motion‐detection simulations. Owing to multi‐V_hold_ spatiotemporal encoding, the proposed RC architecture achieves a 3.98‐fold reduction in device‐array‐level read energy and requires 8.00‐fold fewer trainable synaptic weights than the ANN baseline. These results position oxide 2T memory as a strong candidate for next‐generation non‐von‐Neumann artificial intelligence (AI) hardware.

## Graphical abstract

This work presents a reconfigurable reservoir‐computing platform based on an oxide InSnZnO two‐transistor cell in which a hold bias selects volatile relaxation for the reservoir or quasi‐non‐volatile multilevel storage for the readout. Multi‐level hold‐bias kernel tune relaxation currents to generate diverse time constants, enriching reservoir states and compressing spatiotemporal information. Handwritten digit recognition and motion detection validate scalable, ultralow‐energy operation.

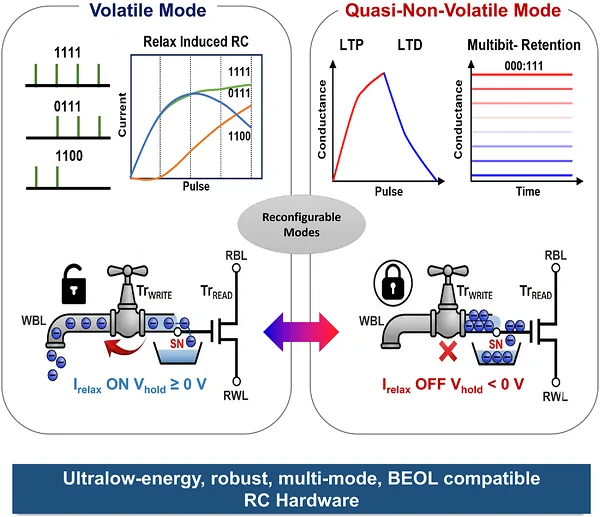

## 1 Introduction

Rapid advances in artificial intelligence (AI) are placing growing demands on conventional computing hardware [1, 2, 3, 4]. As manufacturing technologies continue to scale, transistor dimensions are approaching fundamental physical limits, making further increases in device density increasingly challenging. These constraints drive up fabrication costs and power consumption [5, 6, 7]. At the same time, the von Neumann separation of processing and memory aggravates data‐movement overheads, leading to the well‐known memory bottleneck. To address these challenges, neuromorphic computing architectures have emerged as a promising alternative by co‐locating memory and computation and leveraging brain‐inspired principles for efficient information processing [8, 9]. Among the various neuromorphic approaches, reservoir computing (RC) has gained attention as a particularly attractive framework for temporal and time‐series learning, because it can reduce training complexity by restricting trainable parameters primarily to the readout layer [10, 11].

RC provides an energy‐efficient approach to temporal information processing by exploiting a fixed nonlinear dynamical system, or reservoir, that transforms input sequences into a high‐dimensional state space while restricting trainable parameters primarily to the readout layer [12, 13]. Practical hardware implementation therefore requires two complementary device characteristics: reproducible short‐term dynamics in the reservoir for temporal feature extraction and sufficiently long‐retention analog states in the readout for storing the weight matrix used in vector–matrix multiplication [14, 15]. Physical RC systems based on memristors [16, 17, 18], ferroelectric devices [19, 20, 21], two‐dimensional materials [22, 23], and synaptic transistors [24] have demonstrated diverse approaches to realizing these functions. In many implementations, reservoir dynamics and readout storage are provided by heterogeneous device structures, increasing fabrication and integration complexity [18, 20, 22]. Homogeneous RC architectures in which a common device structure supports both reservoir and readout functionalities have also been demonstrated [25, 26, 27]. However, the resulting hardware benefits have been less extensively quantified from a device‐array perspective, particularly through direct comparisons with conventional ANN architectures under consistent device‐mapping and energy‐accounting conditions. Thus, whether physical RC can simultaneously reduce trainable‐device requirements and device‐array energy consumption remains an important consideration for practical hardware implementation. Operating speed is another critical consideration because slow reservoir dynamics directly increase the time required for temporal‐state evolution and can constrain throughput as data volume and temporal complexity increase. A practical edge‐AI RC platform therefore requires not only low energy consumption, but also fast temporal dynamics, analog uniformity, cycle‐to‐cycle reproducibility, and compatibility with back‐end‐of‐line processing [28].

Oxide‐semiconductor thin‐film transistors based on materials such as InGaZnO and InSnZnO provide an attractive basis for addressing these requirements owing to their ultralow off‐state current, large‐area uniformity, and compatibility with monolithic three‐dimensional integration [29, 30, 31, 32]. These characteristics are particularly advantageous for capacitorless two‐transistor (2T) gain cells, in which the gate capacitance of the read transistor functions as the storage node and the write transistor controls the charge‐transfer pathway. Since the first demonstration of oxide‐channel capacitorless 2T cells [33], this architecture has been extensively investigated as an alternative dynamic random‐access‐memory configuration [34]. Conventional 2T‐memory studies have primarily exploited the ultralow off‐state current of oxide transistors to suppress storage‐node leakage, thereby extending retention for multibit storage [35, 36, 37] and enabling highly scalable memory arrays compatible with monolithic three‐dimensional integration [32, 38, 39]. Although the use of two transistors introduces a larger lateral footprint than RRAM‐based or single‐transistor reservoir elements, recent demonstrations of vertically integrated oxide 2T0C DRAM cells suggest that this area penalty can be substantially alleviated through monolithic three‐dimensional stacking [40, 41, 42, 43].

For reservoir computing, however, the storage‐node leakage pathway can serve a fundamentally different role. Rather than being minimized exclusively for retention, the discharge current can be deliberately regulated to generate controlled charge relaxation and fading‐memory dynamics. In an oxide 2T gain cell, this relaxation is governed by the conduction state of the write transistor, providing a direct electrical means of controlling storage‐node discharge. Such transistor‐controlled relaxation is particularly attractive for physical RC because reproducible temporal‐state evolution is required for reliable encoding of input history. The combination of ultralow off‐state leakage and electrically adjustable write‐transistor conduction therefore provides access to a broad dynamic range, from strongly suppressed charge loss for long‐retention operation to accelerated relaxation for short‐term temporal processing. We refer to the intentionally regulated storage‐node discharge current as the relaxation current, as it directly governs the temporal evolution of the stored charge state.

Controllability of this relaxation process is also important for expanding the computational functionality of a physical reservoir. A reservoir with only a narrow range of intrinsic decay responses provides limited temporal diversity, whereas electrically programmable relaxation dynamics can generate multiple fading‐memory responses from the same device architecture. Distinct relaxation rates can enrich the temporal state space and improve the representation of input sequences without requiring proportional replication of physical reservoir elements [44]. In particular, voltage‐controlled modulation of the relaxation pathway provides a simple route to generating multiple temporal kernels for spatiotemporal tasks such as motion recognition, speech processing, and time‐series analysis [45, 46].

Here, we experimentally characterize this V_hold_‐reconfigurable ITZO 2T platform across volatile and bias‐stabilized quasi‐non‐volatile operating regimes. The operating mode is determined by the voltage bias, V_hold_, applied during the hold step, which regulates the relaxation kinetics of the storage node and enables a retention window spanning from microseconds (volatile reservoir operation) to hours (quasi‐non‐volatile weight storage for the readout). In the reservoir mode, the device exhibits highly robust microsecond‐scale relaxation with endurance exceeding 10^4^ cycles, enabling ultralow reservoir read energy operation of 47.7 fJ/pulse. The read energies reported here are among the lowest reported for analog reservoir‐computing devices in Table S1, highlighting the outstanding energy and speed advantage of the proposed multi‐mode 2T platform. In the readout mode, suppressing the relaxation current yields quasi‐non‐volatile multilevel retention up to 8,604 s, together with robust potentiation/depression over more than 10^4^ cycles and ultralow VMM read energy of 9.92 fJ/pulse. This dual‐mode capability allows both the reservoir and the readout vector–matrix multiplication layer to be implemented using the same device architecture and fabrication process, reducing process complexity and variability. We validate the proposed platform using handwritten‐digit classification and highly compressed motion detection, using measured device characteristics integrated into hardware‐informed simulations. We introduce a multi‐V_hold_ kernel to generate diverse temporal dynamics with programmable relaxation currents, enhancing state richness for spatiotemporal processing and enabling highly compressed‐data motion detection. Quantitatively, V_hold_‐kernel multiplexing expands state richness in the bias domain, enabling spatial compression that reduces the number of trainable synaptic weights by 8.00‐fold and the estimated device‐array‐level read energy by 3.98‐fold relative to the ANN baseline for the evaluated motion‐classification task. The average reservoir read energy was estimated to be 7.13 pJ per motion operation.

Figure S1 benchmarks our platform against other vector–matrix multiplication (VMM) cores in terms of process maturity, integration density, process simplicity, and process reliability, highlighting its distinctive advantage. Overall, this work proposes the ITZO‐based 2T cell as a practical solution for energy‐efficient, robust, cost‐effective, mass‐producible and monolithic‐3D‐compatible reservoir computing hardware.

## 2 Results

### 2.1 V_hold_‐Reconfigurable Oxide 2T Gain Cell for Multi‐Mode Reservoir Computing

Figure 1a,b illustrates that a single 2T cell can be reconfigured into two operating modes by simply adjusting the hold bias, V_hold_. When V_hold_ is raised above 0 V (> V_TH_), charge stored at the storage node (SN) is allowed to discharge through a controlled pathway toward the write transistor (write TR), producing volatile relaxation dynamics. In contrast, when V_hold_ is set below 0 V (< V_TH_), the discharge pathway is suppressed, enabling the SN to retain the written charge state and thereby exhibiting bias‐stabilized quasi‐non‐volatile behavior. Time‐varying input signals are processed by a physical reservoir operated in the volatile regime, where the controlled discharge naturally generates fading memory. The readout layer, in turn, exploits the quasi‐non‐volatile regime to store multi‐bit weights with sufficiently long retention for vector–matrix multiplication. Figure 1c further illustrates multi‐V_hold_ operation, in which the relaxation rate is modulated electrically through the write‐transistor conduction pathway. This enables multi‐V_hold_ map multiplexing, which increases the separability and richness of reservoir states in the time domain, thereby enabling spatial compression that reduces the required trainable‐weight count and the estimated device‐array‐level read energy of the downstream readout.

**FIGURE 1:**
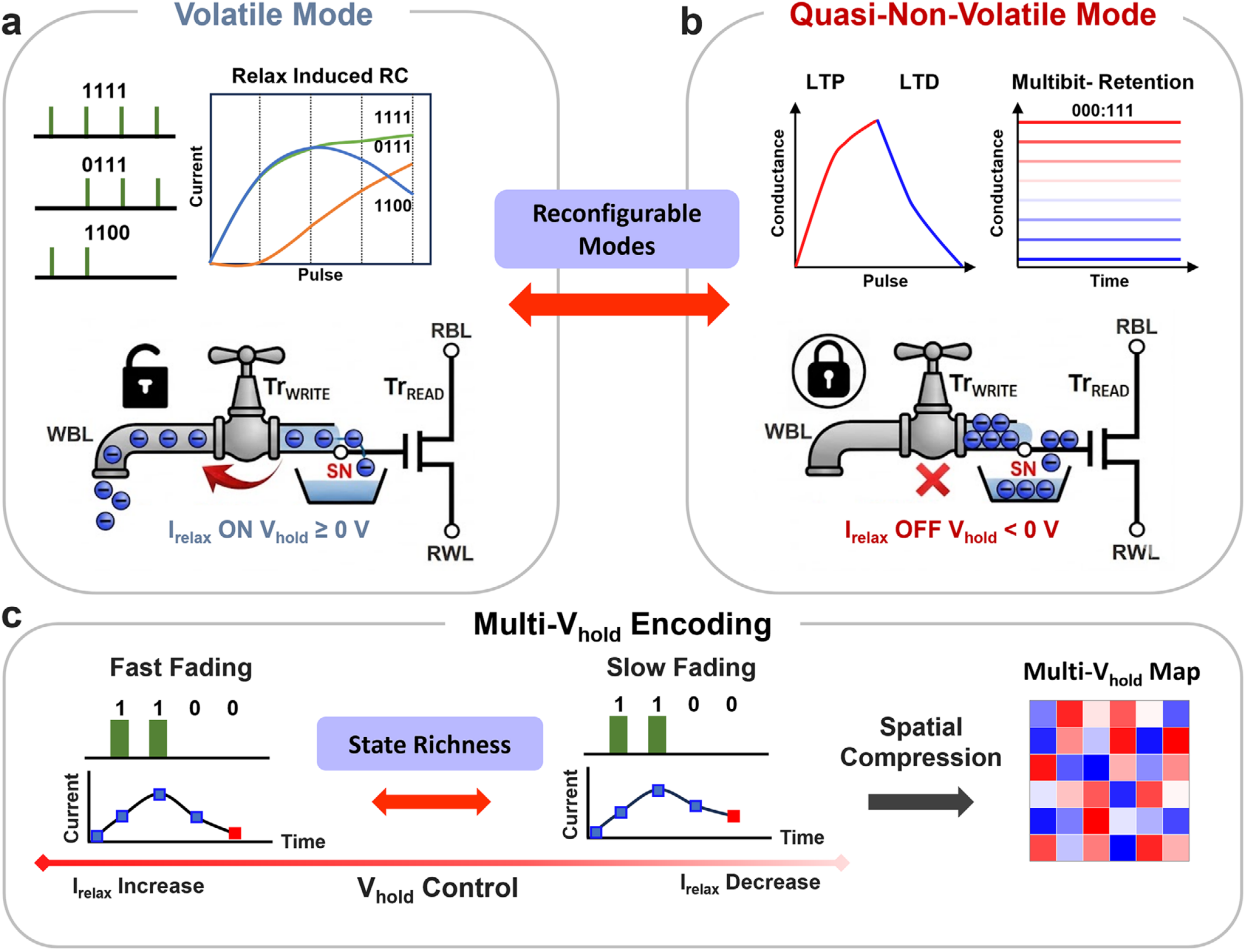
Concept of the reconfigurable oxide 2T platform for multi‐mode reservoir computing. Schematic illustration of a single two‐transistor (2T) cell that can be reconfigured between (a) volatile and (b) quasi‐non‐volatile operation by tuning the hold bias, V_hold_. The hold bias controls charge relaxation at the storage node, thereby determining whether the stored charge is rapidly discharged to generate fading‐memory dynamics or retained to form stable quasi‐non‐volatile memory states. In the volatile mode, the 2T cell provides time‐dependent current relaxation and fading‐memory behavior for use as the physical reservoir. In the quasi‐non‐volatile mode, the same cell supports stable multilevel conductance states that serve as programmable readout weights for vector–matrix multiplication. (c) Concept of multi‐V_hold_ operation, in which electrical modulation of the relaxation pathway generates distinct temporal responses. This bias‐domain kernel multiplexing enriches the reservoir state space and enables spatial compression, thereby reducing the dimensionality and trainable‐weight requirement of the downstream readout.

### 2.2 Quasi‐Non‐Volatile Characteristics of 2T for Readout Network

We first investigated the quasi‐non‐volatile operating characteristics of the proposed 2T memory in Figure 2. The quasi‐non‐volatile mode operation of 2T memory with V_hold_ < 0 V is illustrated in Figure 2a. Because a negative V_hold_ remains applied to suppress storage‐node discharge, this operating regime is referred to as bias‐stabilized quasi‐non‐volatile storage rather than conventional nonvolatile memory [23]. During the write operation, the write TR is turned on by applying V_ON_ to the write word line (WWL), while V_write_ is applied to the write bit line (WBL). Depending on the polarity and magnitude of the applied bias, charges are either injected into or removed from the storage node, thereby modulating the electric field applied to the read transistor (read TR). After programming, the voltage at WWL is switched to V_hold_ to place the device in the hold state, which preserves the stored charge at the storage node. Readout is performed by applying a read bias of 0.1 V to RBL (read bit line) while keeping RWL (read word line) = 0 V. This separates the roles of the two transistors and enables non‐destructive readout, because the programmed charge state at the storage node can be maintained during the sensing operation.

**FIGURE 2:**
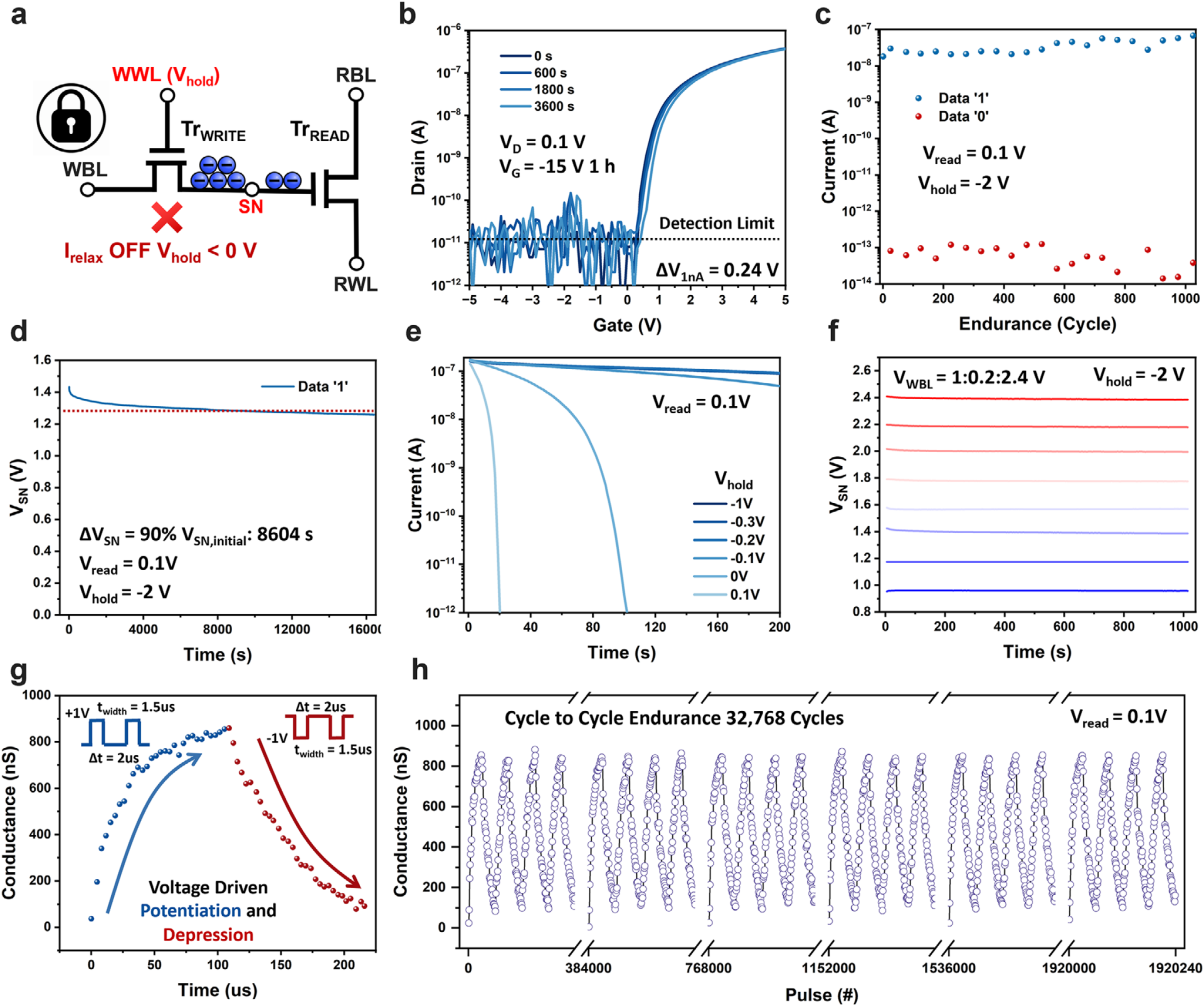
Quasi‐non‐volatile operation, retention programmability, and multi‐level capability of the 2T memory. (a) Quasi‐non‐volatile operation of the 2T memory in the V_hold_ < 0 V regime. (b) Negative‐bias‐stress stability of the oxide TFT, showing a threshold‐voltage shift of ΔV_TH_ = 0.24 V under −15 V for 1 h. (c) Endurance of repeated memory cycling over 2^10^ cycles (displayed with a 25‐cycle interval). (d) Retention of the programmed ‘1’ state under V_hold_ = −2 V, maintaining the state for 8,604 s. (e) Current retention as a function of time under different V_hold_ conditions, showing prolonged retention at negative V_hold_ and progressively faster decay as V_hold_ approaches and exceeds 0 V. (f) Multi‐level quasi‐non‐volatile storage demonstrating 3‐bit retention over 1,000 s, programmed by sweeping WBL = 1.0–2.4 V in 0.2 V steps under otherwise identical bias conditions. (g) Microsecond‐scale potentiation and depression measured using a 4‐point PMU scheme (pulse width 1.5 µs, WBL = 1 V, pulse gap 2 µs), demonstrating fast conductance updates enabled by charge modulation at the storage node. (h) Cycle‐to‐cycle robustness of potentiation/depression over 32 768 cycles.

Bias‐stress stability of the oxide thin‐film transistor (TFT) is summarized in Figure 2b. Under negative bias stress (NBS) of −15 V for 1 h, the threshold voltage shift was ΔV_TH_ = 0.24 V. NBS is particularly critical for 2T operation because the device spends most of its time in the hold state, where a negative bias is applied to WWL to retain the charge at the storage node. Preserving the transfer characteristics under prolonged bias stress is essential for both endurance and retention. If V_TH_ drifts during repeated write/read/hold cycles, the required V_ON_ and V_hold_ must be re‐adjusted, which can lead to write/read/retention failures. The corresponding positive bias stress (PBS) result and gate‐leakage characteristics are provided in Figure S2, showing ΔV_TH_ = 0.34 V under +15 V for 1 h, while the gate leakage remained near the measurement detection limit without any indication of dielectric breakdown. Endurance was evaluated by repeated cycling (write ‘1’ → read ‘1’ → write ‘0’ → read ‘0’) as shown in Figure 2c. Here, one endurance cycle is defined as a complete write ‘1’ → read ‘1’ → write ‘0’ → read ‘0’ sequence. This sequence was repeated 2^10^ cycles (plotted with a 25‐cycle interval). WBL = 1 V, WWL = 4 V, RWL = 0 V, RBL = 0.1 V, V_hold_ = ‐2 V were utilized to write and read. Across cycling, the programmed readout levels remained stable without noticeable degradation, indicating reliable endurance under repeated write/read cycling.

A near‐zero V_TH_ in the oxide transistor is crucial for minimizing the voltage amplitudes required for 2T write, read, and hold operations. Writing requires applying positive bias to WWL while turning on the write TR to inject or remove charge from the storage node. After programming, the write TR must remain in the hold state (negative bias) to minimize leakage through the write path and maintain the stored charge. Because the hold state occupies the largest fraction of the operation timeline, the turn‐off voltages of the write TR strongly impact the overall power consumption. Therefore, careful channel engineering is required, particularly control of the oxygen vacancy concentration in InSnZnO (ITZO), to tune V_TH_ close to zero and minimize the required operating voltages. This approach also reduces the electrical stress applied to the gate dielectric, which is beneficial for device reliability and lifetime. By precisely controlling the oxygen partial pressure during RF sputtering, we achieved a systematic left shift of V_TH_, as summarized in Figure S3. Importantly, this tuning was achieved while maintaining the subthreshold swing and bias‐stress stability, with a reduction in mobility.

Using the optimized near‐zero‐V_TH_ device, we successfully maintained the written ‘1’ state with V_hold_ = −2 V for 8604 s, as shown in Figure 2d. The retention time was defined as the point at which V_SN_ decreased to 90% of its initial value. The measured retention time of 8604 s, corresponding to approximately 2.39 h, enables the programmed readout states to be preserved over a large number of inference operations without continuous reprogramming. Nevertheless, for operation beyond the demonstrated retention window, periodic refresh or weight reprogramming would be required before the stored state falls outside the acceptable read margin. Further lateral scaling may also shorten the retention time because the total storage‐node capacitance decreases as C**_SN_ =** C**_OX_** ×**A**. For a comparable leakage current, a smaller C**_SN_** results in faster storage‐node voltage decay, dV**_SN_**/dt **=** −I**_leak_**/C**_SN_**, thereby increasing the required refresh rate. Retention was evaluated based on the ‘1’ state because retention failure occurred earlier than for the ‘0’ state. The raw readout currents for ‘1’ and ‘0’ prior to conversion into V_SN_ are provided in Figure S4, and the current‐to‐V_SN_ conversion is described in Figure S5 and Note S1. We further examined retention programmability by varying V_hold_ (Figure 2e). When V_hold_ ≤ −0.1 V, the retention time exceeded 100 s, consistent with sufficiently suppressed leakage. As V_hold_ approached 0 V, retention began to decrease gradually, and for V_hold_ ≥ +0.1 V, the retention collapsed rapidly to the sub‐second regime. These results demonstrate that V_hold_ can continuously tune the memory behavior from quasi‐non‐volatile operation (hours) to intentionally volatile operation (sub‐second scale). The corresponding ‘0’ state behavior is shown in Figure S6. The retention measurements in Figure 2d–f used a single 0.2 s write pulse to sufficiently charge the effective storage node before long‐term monitoring. For practical device‐to‐device volatility control, maintaining stable transistor parameters across devices is essential to ensure that the transition voltage between quasi‐non‐volatile and volatile modes remains within a narrow, controllable range. Figure S7 summarizes the distributions of V_TH_, mobility, and subthreshold swing for 20 devices, confirming device‐to‐device uniformity required for reproducible retention tuning. Multi‐level quasi‐non‐volatile storage was demonstrated in Figure 2f, which shows 3‐bit retention over 1000 s. Multi‐bit states were programmed by sweeping WBL from 1.0 to 2.4 V in 0.2 V steps, while keeping the remaining voltage conditions identical to the baseline operation. The resulting states are clearly separated, supporting quasi‐non‐volatile multi‐level retention suitable for vector–matrix applications in the readout layer of physical reservoir computing. The current‐level data of Figure 2f are shown in Figure S8.

Finally, we characterized synaptic updates using pulse measurements. Figure 2g shows potentiation and depression in the microsecond regime measured using a 4‐point pulse modulation unit (PMU) configuration. Owing to rapid electron transport associated with charge modulation at the storage node, the device enables fast conductance updates. The pulse width was 1.5 µs, pulse amplitude on WBL was 1 V, and the pulse gap was 2 µs, as indicated in the inset of Figure 2g. In Figure 2h, cycle‐to‐cycle robustness was confirmed by applying 32,768 potentiation/depression cycles, demonstrating stable conductance modulation that is critical for reliable vector–matrix multiplication in a readout network. Here, one cycle denotes one complete potentiation/depression sequence under the conditions shown in Figure 2g, which was repeated 32 768 times.

### 2.3 Volatile Characteristics of 2T for Physical Reservoir

We next investigated the volatile operating characteristics of the same 2T device for physical reservoir computing. Figure 3a illustrates the volatile mode, in which V_hold_ is set above 0 V to intentionally allow charge relaxation from the storage node toward WBL through the write TR path.

**FIGURE 3:**
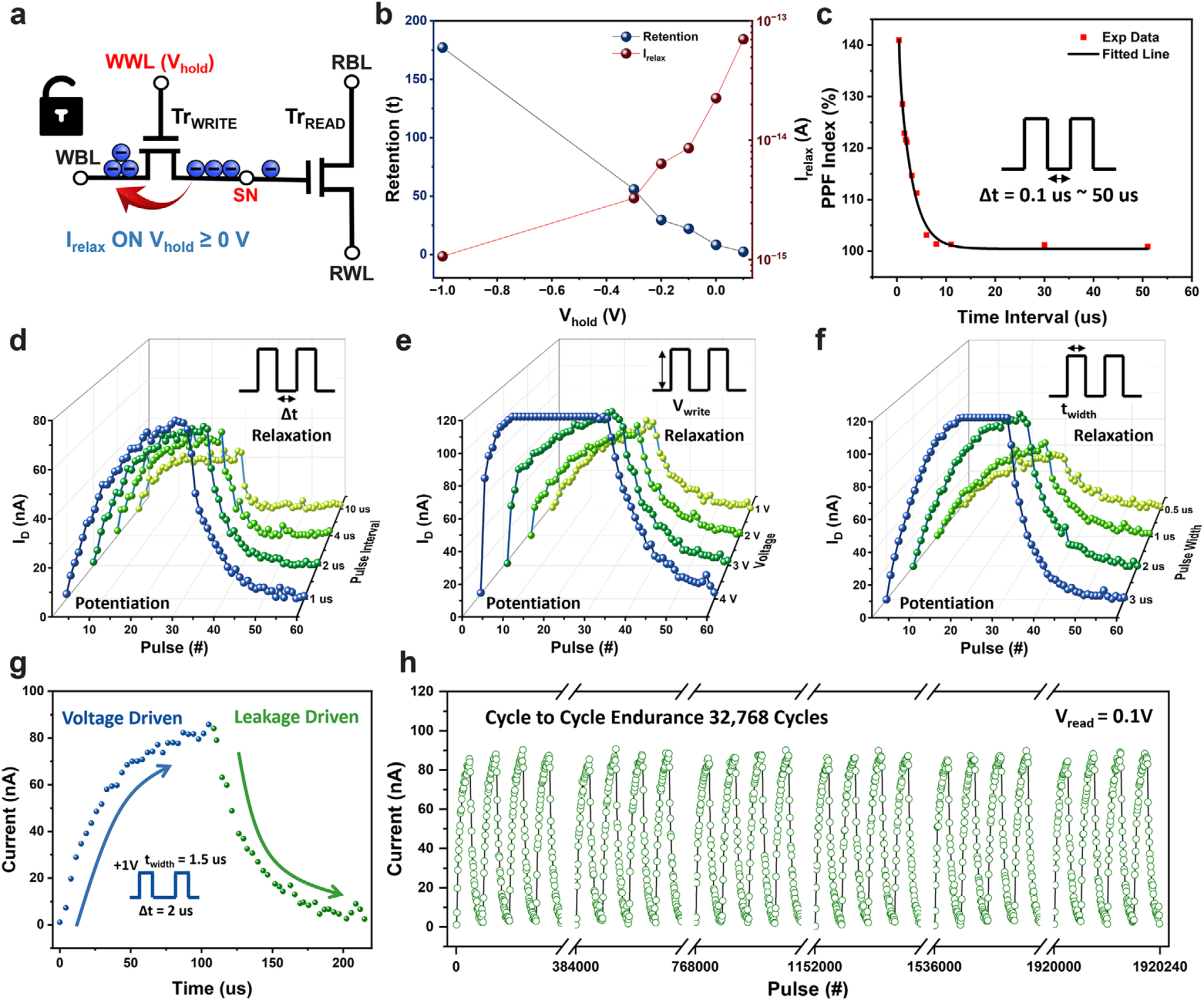
Volatile operation and leakage‐driven short‐term dynamics of the 2T device. (a) Schematic of the volatile mode, where V_hold_ > 0 V intentionally opens a leakage path. (b) V_hold_‐dependent retention time (left axis) and effective storage‐node relaxation current (right axis), both derived directly from the measured V_SN_ trajectories. The retention time is defined as the time required for V_SN_ to decrease to 90% of its initial value. (c) Paired‐pulse facilitation (PPF) as a function of inter‐pulse interval. (d) Potentiation followed by relaxation under V_hold_ = 0 V with fixed pulse width (2 µs) and amplitude (WBL = 1 V) while varying the inter‐pulse interval (1–10 µs). (e) Potentiation and subsequent relaxation with fixed pulse width (1 µs) and interval (2 µs) while varying amplitude (WBL = 1–4 V). (f) Potentiation and subsequent leakage‐driven decay with fixed amplitude (WBL = 1 V) and interval (2 µs) while varying pulse width (0.5–3 µs). (g) Optimized volatile operating condition (WBL = 1 V, pulse width 1.5 µs, interval 2 µs) showing potentiation and controlled relaxation. (h) Cycle‐to‐cycle robustness of the optimized volatile operation over 32 768 cycles under the same conditions as in (g) (WBL = 1 V, pulse width = 1.5 µs, and interval = 2 µs).

To directly quantify the V_hold_‐dependent storage‐node relaxation, we analyzed the experimentally measured V_SN_ trajectories rather than relying on the write‐transistor current measured under a fixed external drain bias. The retention time was defined as the time required for V_SN_ to decrease to 90% of its initial value, and the corresponding effective relaxation current was calculated as:

(1) $$\begin{array}{c} I_{relax}=C_{SN}\times \;\left(\frac{0.1V_{SN,0}}{t_{ret}}\right) \end{array}$$

where C_SN_ is the storage‐node capacitance. As shown in Figure 3b, increasing V_hold_ systematically shortens the retention time and increases I_relax_. Over the directly evaluated range from V_hold_ = −0.3 to +0.1 V, I_relax_ increases from 3.26 × 10^−15^ to 7.01 × 10^−14^ A, directly demonstrating strong electrical control of the storage‐node relaxation by V_hold_.

The extracted I_relax_ represents the net storage‐node charge‐loss rate rather than a single microscopic leakage current. In addition to subthreshold conduction through the write transistor, gate leakage, tunneling‐related leakage, and parasitic‐capacitance‐induced charge redistribution can contribute to the measured V_SN_ evolution [47]. These contributions can depend differently on V_hold_, V_SN_, and the instantaneous transistor terminal voltages. Furthermore, parasitic capacitances coupled to the storage node can induce an abrupt V_SN_ change during transitions between write/hold and read conditions [37, 48, 49]. Therefore, both transistor leakage characteristics and device geometry must be considered when analyzing the retention behavior of a 2T gain cell.

For reference, the independently measured write‐transistor current at V_DS_ = 0.1 V is provided in Figure S9a. Because this static measurement imposes a fixed drain bias, whereas the storage node remains floating and continuously evolves during retention, it is used only as a qualitative indicator of the gate‐dependent conduction tendency and is not quantitatively equated with I_relax_. The measured ΔV_SN_(t) trajectories under different Vhold values are provided in Figure S9b.

Volatility was further evaluated using PPF, as shown in Figure 3c. PPF probes short‐term dynamics by applying two consecutive pulses with a controlled inter‐pulse interval. When the inter‐pulse interval was reduced below 10 µs, the PPF ratio increased, reaching ∼140%, indicating strong short‐term facilitation consistent with volatile/decaying internal states.

Based on these observations, we examined voltage‐driven potentiation followed by leakage‐driven current decay under fixed conditions of V_hold_ = 0 V and a compliance current of 114.7 nA. In Figure 3d, the pulse width was fixed at 2 µs and the pulse amplitude (WBL) at 1 V, while the inter‐pulse interval was varied among 1, 2, 4, and 10 µs. Shorter inter‐pulse intervals produced higher current accumulation. After 30 potentiation pulses, we applied 30 relaxation pulses, during which only the read operation was performed (0.1 V bias at RBL with RWL = 0 V), allowing the conductance to relax primarily via leakage. A similar potentiation and decay sequence is shown in Figure 3e, where the pulse width was fixed at 1 µs, the inter‐pulse interval at 2 µs, and the pulse amplitude (WBL) was swept from 1 to 4 V. Again, 30 potentiation pulses were followed by 30 leakage‐driven read‐only pulses, demonstrating systematic modulation of the potentiation level and subsequent decay behavior with programming amplitude. In Figure 3f, the amplitude was fixed at 1 V and the inter‐pulse interval at 2 µs, while the pulse width varied from 0.5 to 3 µs.

Across these parametric sweeps (interval, amplitude, and width), we identified an operating condition that provides stable potentiation and well‐controlled leakage‐driven decay. The optimized pulse scheme is summarized in Figure 3g, using WBL = 1 V, pulse width 1.5 µs, and inter‐pulse interval 2 µs. To evaluate cycle‐to‐cycle robustness, the same optimized condition used in Figure 3g (WBL = 1 V, pulse width = 1.5 µs, and inter‐pulse interval = 2 µs) was applied, with one cycle defined as 30 voltage‐driven potentiation pulses followed by 30 leakage‐driven relaxation steps. This complete cycle was repeated 32,768 times, as shown in Figure 3h. The device maintained consistent potentiation and decay characteristics with minimal drift over extended cycling, confirming the stability of the volatile operation in the 2T device. The detailed voltage waveforms applied to WBL and WWL for the optimized condition are provided in Figure S10.

Importantly, reproducible temporal decay is essential for physical RC because the evolving reservoir state directly encodes input history. Fluctuation or drift in the decay dynamics can therefore distort the reservoir states and reduce reliability. The 2T architecture addresses this issue by regulating storage‐node discharge through V_hold_‐dependent write‐transistor conduction, providing an electrically controlled and reproducible relaxation pathway. The consistent relaxation trajectories maintained over 32,768 cycles support stable temporal‐state generation under repeated operation. Temperature dependence and long‐term aging remain important topics for future evaluation.

### 2.4 Ultralow‐Energy, Fast, Robust Reservoir for Handwritten Digit Recognition

Using the volatile device characteristics established above, we evaluated 4‐bit temporal current responses for physical‐reservoir operation in MNIST handwritten‐digit recognition (Figure 4). Figure 4a shows the 4‐bit physical‐reservoir current response. A sequence of four input pulses encoding binary patterns from 0000 to 1111 was applied with a pulse width of 1 µs, an inter‐pulse interval of 0.8 µs, and an amplitude of WBL = 1 V. Under these conditions, the device current rises and relaxes nonlinearly within each pulse window, yielding 16 clearly distinguishable output states evaluated at the fourth pulse. Notably, the required pulse timescale is the shortest reported among the physical‐reservoir demonstrations compared in Table S1. This is because the volatility in our 2T platform is governed by electronic charge leakage from the storage node once the leakage path is opened, enabling rapid state evolution. In contrast, reservoir implementations relying on ionic/vacancy migration (e.g., in many RRAM‐based systems) typically exhibit slower intrinsic dynamics, leading to longer characteristic time scales. This time scale directly impacts the overall computation latency in a fully integrated reservoir‐computing system, because the state update and readout can be started after the reservoir's response.

**FIGURE 4:**
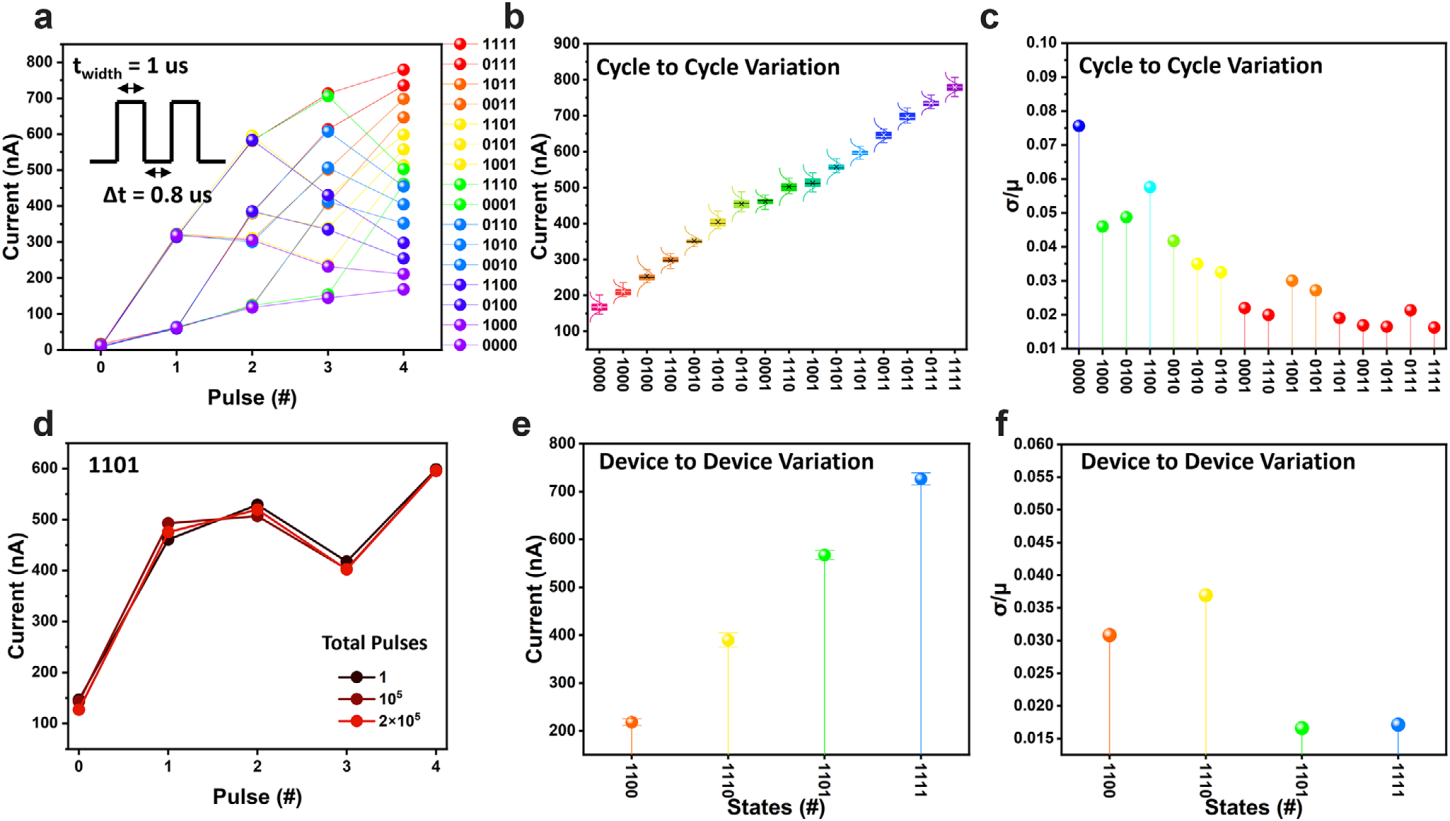
4‐bit physical reservoir states and robustness of volatile operation. (a) 4‐bit reservoir‐current states generated by applying four‐pulse input sequences encoding all binary combinations from 0000 to 1111. Measurements were performed using 1 µs input pulses separated by 0.8 µs at WBL = 1 V, with a compliance current of 1.13 µA. The output current, read at the fourth pulse, forms 16 distinct volatile states determined by the preceding pulse sequence, evidencing the capability of the reservoir to map temporal input history into distinguishable current levels. (b) Statistical distributions of the 16 reservoir states obtained from 20 repeated measurements on the same device (*n* = 20); data are presented as mean ± standard deviation (SD). (c) Corresponding coefficients of variation (CV = σ/µ) calculated from the same 20 repeated measurements. (d) Endurance test of a representative state (1101) over 2 × 10^5^ repeated cycles. (e) Device‐to‐device distributions of four representative reservoir states measured across 12 devices from a single fabrication batch (*n* = 12 devices); data are presented as mean ± SD. (f) Corresponding device‐to‐device CV values.

The compliance current was set to 1.13 µA to expand the available dynamic range for multi‐bit operation. The four blue points highlighted in the red box, as illustrated in Figure S11, indicate the operating points used to evaluate the 4‐bit reservoir performance. An example voltage waveform for the 1110 state is provided in Figure S12. To assess cycle‐to‐cycle variability, the full set of 0000–1111 pulse sequences was repeated over 20 trials, as summarized in Figure 4b. The mean and dispersion of the current outputs confirm that the 16 states remain separable across repeated measurements; the complete dataset for cycle‐to‐cycle variation is provided in Figure S13. State reliability was quantified using the coefficient of variation, σ/µ, as shown in Figure 4c. All states exhibited σ/µ < 0.08, indicating stable and reproducible multi‐level outputs. The 0000 state shows a relatively larger σ/µ due to its low absolute current level, where measurement noise and small fluctuations contribute more strongly after normalization.

In Figure 4d, endurance of volatile multi‐level operation was further verified by repeatedly applying the 1101 state for 2 × 10^5^ cycles, during which the output current remained stable. Device‐to‐device reproducibility was evaluated across 12 devices in Figure 4e, showing that the 4 output states remain distinguishable across devices. The corresponding device‐to‐device σ/µ values are summarized in Figure 4f, confirming minimal dispersion in multi‐state volatile behavior. The complete dataset for device‐to‐device variation is provided in Figure S14. Overall, these results demonstrate that the proposed 2T platform provides robust and repeatable multi‐level volatile dynamics, which is essential for stable, practical physical reservoir computing.

Using the experimentally measured volatile reservoir responses (Figure 4b) and quasi‐non‐volatile conductance states (Figure 2g), we evaluated MNIST digit recognition using a hardware‐informed RC architecture based on the measured 2T device characteristics. Figure 5a illustrates the encoding procedure using the digit “5” as a representative example. Each 20 × 20 binary image was divided into 100 non‐overlapping 2 × 2 patches. Within each patch, the four binary pixels were temporally encoded in a predefined Z‐shaped order, producing one of 16 possible 4‐bit input patterns (0000–1111). The corresponding 4‐bit pulse trains were applied to the volatile 2T physical reservoir as depicted in Figure 5b. Each encoded patch was driven by a temporal input sequence with a total duration of 7.2 µs (pulse width 1 µs, interval 0.8 µs, four pulses), generating a reservoir output state that was subsequently processed by a simulated 100 × 10 quasi‐non‐volatile 2T readout array to produce the final digit prediction. A comprehensive description of the simulation procedure and parameter settings is provided in the Methods section.

**FIGURE 5:**
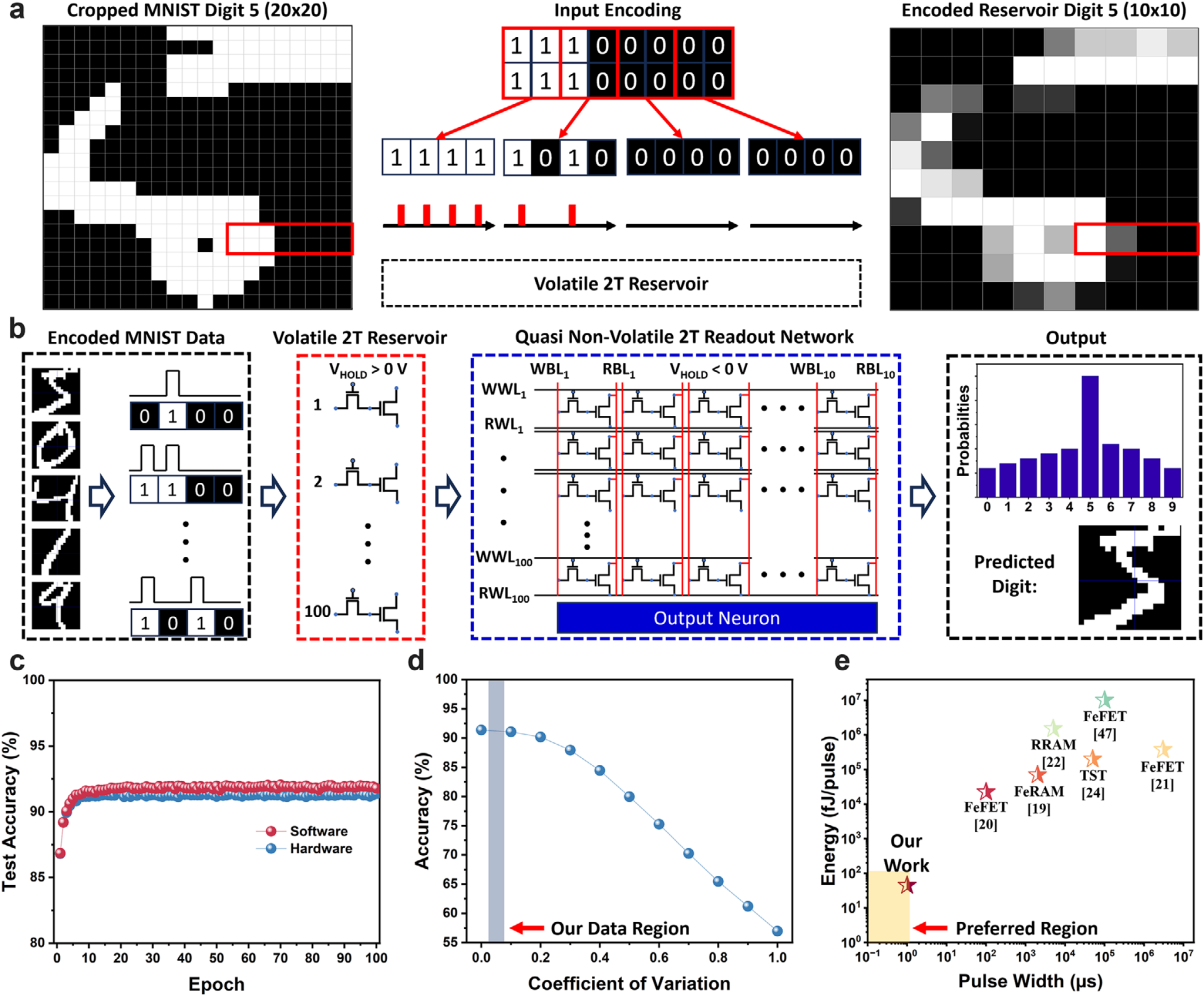
MNIST classification using a 2T‐based volatile reservoir and quasi‐non‐volatile readout network. (a) Input representation for MNIST inference: binarized and cropped digit images are mapped to 20 × 20 binary images and partitioned into 10 × 10 non‐overlapping 2 × 2 patches, forming 100 reservoir inputs encoded by 4‐bit pulse patterns. (b) Hardware‐informed inference architecture combining the volatile 2T physical reservoir and a simulated 100×10 quasi‐non‐volatile 2T readout array that produces the final digit classification from reservoir states. (c) Test accuracy versus training epochs comparing hardware‐informed evaluation and a software baseline. (d) Robustness analysis of classification accuracy against injected Gaussian noise using 1000 independent Monte Carlo trials at each evaluated noise condition (*n* = 1000 trials). Gaussian noise was parameterized using the experimentally measured reservoir‐current variability, and CV = σ/µ was swept from 0 to 1. The highlighted region indicates our data's σ/µ region. (e) Read energy and pulse‐width comparison with other state‐of‐the‐art reservoir computing systems. The yellow box highlights the low‐energy, short‐pulse‐width region.

Figure 5c summarizes the classification performance after 100 training epochs. The final test accuracy reached 91.37% for the hardware‐informed evaluation (blue) and 91.79% for the software baseline (red). Here, the “hardware” result incorporates measured electrical characteristics from both the volatile reservoir and the quasi‐non‐volatile readout devices, whereas the “software” result uses only the physical‐reservoir response while excluding quasi‐non‐volatile readout‐device non‐idealities. The minimal accuracy reduction indicates that introducing the quasi‐non‐volatile readout network does not significantly degrade performance, supporting the practical feasibility of a 2T‐based hardware architecture. The confusion matrix and training loss over the training epochs are shown in Figure S15.

System robustness against device non‐idealities was evaluated using a Monte Carlo framework with 1,000 independent trials. In each trial, Gaussian noise was injected into the reservoir current responses to emulate cycle‐to‐cycle variability:

(2) $$\begin{array}{c} I_{noisy}=max(I_{avg}+N\left(0,\sigma^{2}),0\right) \end{array}$$

using σ derived from experimental measurements. We further quantified tolerance to stochasticity by sweeping the coefficient of variation (CV = σ/µ) from 0.0 to 1.0 and tracking classification accuracy in Figure 5d. (see Figure 4c,f for the experimentally measured σ/µ) This analysis established the noise‐margin curve of the system and enabled direct comparison with the experimentally measured CV of our devices. The high‐dimensional temporal mapping provided by the 2T reservoir preserved stable accuracy across substantial noise levels (blue region in Figure 5d), confirming reliable training and inference under realistic hardware variability.

Energy consumption was evaluated under the operating conditions used in this work. The estimated read energies were only 47.7 fJ per pulse for the physical reservoir and 9.92 fJ per VMM read pulse for a single quasi‐non‐volatile 2T cell, highlighting the energy efficiency of the proposed platform (see Note S2, for the energy calculation procedure). These read‐energy values, together with the microsecond pulse width, place the device in a favorable energy–speed regime reported for comparable analogue hardware reservoir‐computing systems, underscoring the strong technological competitiveness of our unified 2T architecture as depicted in Figure 5e (see Table S1, for comparison with state‐of‐the‐art works) [19, 20, 21, 22, 24, 50, 51, 52]. For 10 000–30 000 pulses, the estimated reservoir read energy based on the experimentally obtained 47.7 fJ per pulse corresponds to approximately 0.48–1.43 nJ. These estimates suggest that, if pulse count and peripheral overhead are properly controlled, such a reservoir can provide not only device‐level efficiency but also a favorable device‐level read‐energy/speed regime over conventional ANN hardware. The array‐level read energy comparison between RC and ANN is further evaluated in Figure 6f.

**FIGURE 6:**
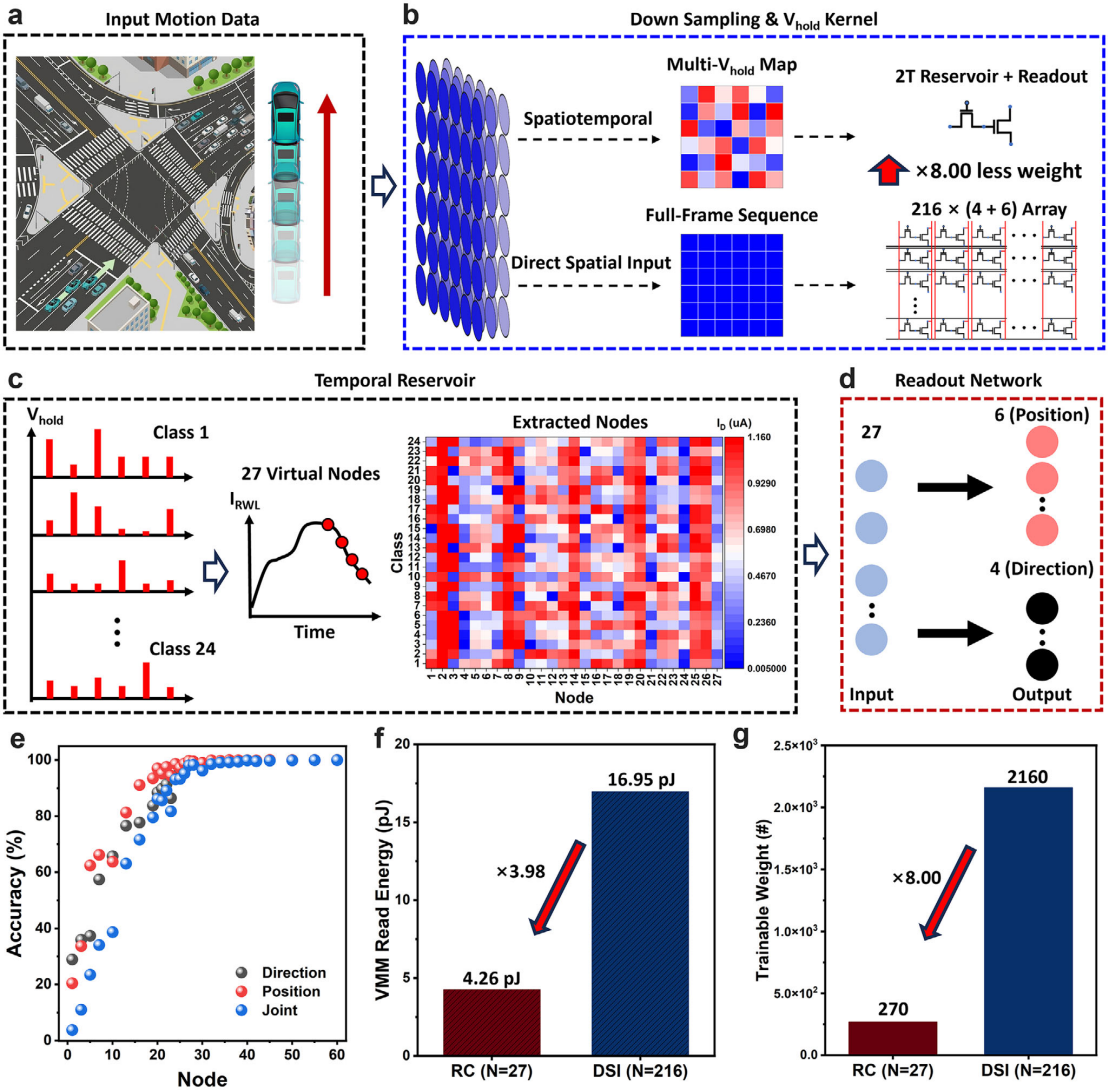
Multi‐V_hold_ spatiotemporal encoding for highly compressed motion detection using a single 2T reservoir. (a) Representative motion‐detection scenario illustrating a practical application of the proposed spatiotemporal encoding scheme. (b) Concept of data compression based on the multi‐V_hold_ kernel response. A multi‐V_hold_ map generates diverse temporal dynamics, allowing a single 2T reservoir to encode a 6 × 6 frame sequence into a compact temporal representation with reduced readout dimensionality. (c) Schematic of the 24‐class reservoir configuration constructed using multi‐V_hold_ encoding. Each motion corresponds to one of the 24 motion classes, and virtual nodes are uniformly extracted from the measured temporal responses. Each response represents the mean of 100 repeated current measurements on the same device (*n* = 100 measurements per class). (d) Structure of the readout network (27 × 6 for position, 27 × 4 for direction) used for motion classification. (e) Classification accuracy as a function of the number of reservoir nodes for position, direction, and joint classification. Joint accuracy denotes the fraction of samples for which both position and direction are correctly classified. (f) Comparison of the estimated VMM read energy between the 27‐dimensional reservoir representation and the 216‐dimensional direct spatial input under the same linear classifier. (g) Comparison of the number of trainable weights between the RC readout (*n* = 27) and the direct spatial input (DSI) (*n* = 216).

It is worth noting that faster operation is feasible by increasing the effective channel mobility and operating at higher current levels; however, such acceleration would come with a trade‐off of increased power and energy consumption, since the switching energy scales with the applied voltage, current level, and pulse width. In addition, in oxide TFT‐based 2T operation, higher mobility is often accompanied by a negative shift in V_TH_ [53], which can impact the V_hold_ margin required for reliable charge storage. A more negatively shifted V_TH_ typically necessitates a larger magnitude hold bias to maintain the stored charge state, which increases long‐term gate stress during the hold period and can raise the standby energy consumption. Therefore, optimizing mobility for speed must be balanced against V_TH_ placement and hold‐bias stress to achieve an overall energy‐efficient and reliable 2T reservoir‐computing system.

### 2.5 Multi‐V_hold_ Spatiotemporal Encoding for Highly Compressed, Ultralow‐Energy Motion Detection

In Figure 6, we demonstrate a spatial‐compression strategy for motion detection based on multi‐V_hold_ spatiotemporal encoding, in which V_hold_ is tuned from 0 to 2 V to access multi‐mode operation. In the earlier sections, V_hold_ was used mainly in two discrete regimes (quasi‐non‐volatile and volatile). Here, we extend V_hold_ to a multi‐level space to diversify decay dynamics effectively by implementing a set of programmable temporal kernels. By feeding input signals into these kernels, the reservoir generates high‐dimensional spatiotemporal features within the bias‐time domain. Importantly, this kernel‐based encoding enables spatial compression by effectively converting spatial features into trajectory‐dependent temporal signatures. High‐dimensional information is extracted from the time domain rather than through the physical duplication of devices, reducing the need for proportional scaling of the number of physical reservoir units.

Figure 6a illustrates a representative motion‐detection scenario (vehicles moving through an intersection), highlighting a practical application of the proposed approach. The core concept is shown in Figure 6b, where consecutive 6 × 6 binary frames are encoded using a multi‐V_hold_ kernel map. In conventional frame‐difference‐based motion detection, pixel‐wise comparisons between f(t−1) and f(t) are performed for every pixel, leading to pixel‐parallel hardware scaling. By contrast, our reservoir approach exploits distinct V_hold_‐dependent relaxation responses, such that spatial information across frames is converted into trajectory‐dependent temporal signatures. As a representative example, when an object moves upward from column 3 (Video S1), the corresponding V_hold_ pattern in the kernel map (Figure S16) is applied to WWL during the hold step after writing. This multi‐V_hold_ spatiotemporal encoding generates motion‐specific relaxation traces that enrich the reservoir states. As a result, the full 6 × 6 frame sequence can be encoded by a single 2T reservoir device, enabling spatial compression of the readout input.

Figure 6c summarizes the multi‐V_hold_ reservoir architecture used for classification. A total of 24 classes were defined, corresponding to 24 output motions (four motion directions and six position indices). For each channel, the measured current response was uniformly discretized to generate a set of virtual nodes that serve as a high‐dimensional feature vector for the readout network. The virtual nodes extracted from the mean of 100 repeated current measurements across the 24 classes are also shown in Figure 6c.

Motion classification was performed using separate ridge regression‐based linear readouts that directly map the virtual reservoir states to six position and four direction classes (Figure 6d). To investigate how reservoir dimensionality affects classification performance, the number of virtual nodes (N) was varied. The direction, position, and joint direction‐position accuracies increased with N, with the joint accuracy progressively approaching the individual direction and position accuracies (Figure 6e). Based on the joint validation performance across the data partitions, *n* = 27 was selected for subsequent analysis. At *n* = 27, the physical reservoir provided a temporal representation that could be directly classified using linear readouts, achieving test accuracies of 98.40% ± 1.73%, 99.68% ± 0.36%, and 98.09% ± 1.78% for direction, position, and joint direction‐position classification, respectively, over 100 randomized data partitions. The corresponding joint direction‐position confusion matrix across the 24 classes is presented in Figure S17. To further examine how reservoir dimensionality relates to readout architecture and computational requirements, ridge regression, softmax regression, and single‐hidden‐layer multilayer perceptron (MLP) readouts were compared across different reservoir dimensions (Figure S18). Under a 95% joint‐accuracy criterion, the softmax regression and MLP readouts reached the target performance at smaller reservoir dimensions than ridge regression, while requiring iterative training and, for the MLP, an additional hidden layer. Direct spatial and frame‐difference representations were also evaluated across ridge regression, softmax regression, and MLP readouts with varying hidden‐layer dimensions, providing additional non‐reservoir baselines for the defined motion task (Figure S19). This demonstration shows that the V_hold_‐dependent device dynamics can encode spatially distributed motion sequences into temporal reservoir states that preserve information on both motion direction and spatial position, providing a basis for further evaluation under more diverse motion trajectories, object characteristics, and input conditions.

Next, the computational benefit of the reservoir‐based dimensionality reduction was evaluated in terms of the VMM read energy required for the subsequent readout operation. In Figure 6f, the 27‐dimensional reservoir representation was compared with the 216‐dimensional direct spatial input under the same linear classifier. With the trained readout weights mapped onto the experimentally measured conductance states, the VMM read energy was 4.26 pJ for the reservoir representation and 16.95 pJ for the direct spatial input, corresponding to a 3.98‐fold reduction.

The compact reservoir representation also reduces the hardware requirement of the subsequent readout. With separate six‐class position and four‐class direction readouts, the 27‐dimensional reservoir representation requires 270 trainable weights, compared with 2,160 weights for the 216‐dimensional direct spatial input, corresponding to an 8.00‐fold reduction in the number of trainable weights (Figure 6g). Under the same differential conductance mapping, the reduced number of trainable weights proportionally decreases the number of conductance cells required to implement the readout array. This reduction is achieved by trading spatial hardware replication for temporal encoding, which is a central advantage of temporal reservoir computing. In the evaluated task, the 2T reservoir generates 27 virtual‐node current features that provide sufficient temporal‐state dimensionality to distinguish among the 24 motion classes. This compact temporal representation enables the use of a single physical reservoir while reducing the size and estimated device‐array read energy of the downstream readout. Although sequential processing introduces latency, the microsecond‐scale electronic charge‐relaxation dynamics help limit this overhead. Because both RC and ANN systems require input‐encoding and interface circuitry, the principal benefit is the compact feature representation delivered to the trainable array. For higher‐dimensional inputs, block‐parallel temporal encoding using multiple 2T reservoirs could provide a scalable balance between throughput and hardware efficiency.

The average read energy consumption of the physical reservoir was estimated to be 7.13 pJ per operation, with the detailed calculation provided in Figure S20. Peripheral and data‐handling contributions are excluded from both representations to ensure a consistent device‐array‐level accounting boundary. The simulation method, energy calculations, and trainable‐weight calculations are detailed in Notes S3 and S4, and Table S2, respectively.

Thus, the multi‐V_hold_ spatiotemporal encoding transforms the original spatial motion sequence into a compact temporal representation that remains linearly separable, reducing the computational requirements of the subsequent readout in terms of both energy consumption and hardware resources.

## 3 Conclusion

We demonstrated V_hold_‐controlled electrical reconfiguration of storage‐node charge relaxation in an ITZO‐based 2T gain‐cell architecture, enabling identical cells fabricated through the same process to serve either as volatile reservoir elements or as bias‐stabilized quasi‐non‐volatile analog readout elements. In the volatile regime, charge‐controlled relaxation provides highly uniform microsecond‐scale fading memory, whereas in the quasi‐non‐volatile regime the same 2T cell enables multilevel weight storage with retention up to 8604 s. The relaxation dynamics originate from controlled charge loss rather than stochastic filament formation, yielding excellent cycle‐to‐cycle and device‐to‐device uniformity together with low device‐level read energy (47.7 fJ/pulse for reservoir read and 9.92 fJ/pulse for VMM read), fast reservoir operation and endurance exceeding 10^4^ cycles in both modes. We further introduced multi‐V_hold_ spatiotemporal encoding to continuously program relaxation currents and generate a family of decay kernels with distinct effective relaxation dynamics. This bias‐domain kernel multiplexing expands reservoir state richness without spatial replication, enabling spatial compression that reduces weight count and improves energy scaling for time‐varying workloads. The concept was validated using handwritten‐digit recognition (91.37% test accuracy) as a static task and highly compressed motion detection as a dynamic task. For motion detection, the RC implementation achieved a 3.98‐fold reduction in estimated device‐array‐level read energy and required 8.00‐fold fewer trainable synaptic weights than the ANN baseline. The corresponding average reservoir read energy was estimated to be 7.13 pJ. Overall, these results show that the ITZO‐based reconfigurable 2T platform offers a simple and practical route to reservoir‐computing hardware with low energy consumption, fast operation, and robust multi‐mode behavior.

## 4 Experimental Section

### 4.1 Device Fabrication

Figure S21 shows the self‐aligned top‐gate (SATG) device architecture, and Figure S22 summarizes the fabrication flow of the ITZO SATG TFT. The devices were fabricated on Si substrates with a 300‐nm thermally grown SiO_2_ layer. A 20‐nm amorphous ITZO film was deposited by RF sputtering, patterned by maskless lithography, and wet‐etched using 2.5% diluted HCl. The patterned ITZO was then annealed in air at 270°C. A gate stack consisting of 41‐nm SiO_2_, 4‐nm Al_2_O_3_, and 65‐nm W was subsequently formed by PECVD, PEALD, and DC sputtering, respectively. After gate definition, the W gate was wet‐etched, and the photoresist was removed by acetone sonication. The underlying SiO_2_/Al_2_O_3_ was then dry‐etched along the gate pattern using ICP‐RIE to define the self‐aligned structure. The devices were annealed again in air at 300°C, followed by Ar‐plasma treatment in ICP‐RIE to metallize the ITZO channel and form n^+^‐ITZO source/drain regions. Source and drain areas were defined by maskless lithography, a 50‐nm W layer was deposited, and lift‐off patterning was used to complete the source/drain electrodes. The final device had a channel length of 20 µm and a channel width of 40 µm. Figure S23 shows the TEM side profile of SATG along with EDS profile, consisting of the channel, gate insulator, and gate.

### 4.2 Electrical Measurement

The electrical characteristics, including the *I*–*V* curves and quasi‐non‐volatile memory behavior, were measured using a Keithley 4200 Semiconductor Characterization System in a four‐probe configuration, consistent with the four‐terminal operation of the 2T device. Pulse‐based measurements were performed using two Keithley 4225‐PMU pulse‐measurement units in combination with two 4225‐RPM remote amplifier/switch modules to enable four‐point pulsed operation. All measurements were conducted under vacuum at 30 mTorr.

### 4.3 Structure and Material Analysis

The cross‐sectional image was investigated using JEM‐F200 (JEOL).

### 4.4 MNIST Handwritten Digit Recognition Simulation

(1) Preprocessing of the Handwritten Digits Dataset: To optimize the input dimensionality for the hardware‐based reservoir system and enhance computational efficiency, the original MNIST handwritten digit images underwent a multi‐step preprocessing sequence. Initially, the grayscale images were transformed into a binary format using a fixed threshold of 0.5. This binarization step is critical to ensure robust signal differentiation against hardware‐induced noise and to simplify the pulse‐encoding logic required for the physical reservoirs. Subsequently, a global tight cropping scheme was implemented to eliminate superfluous border regions and inactive background pixels as in Figure S24. The dimensions for the system were determined by a survey of the entire training dataset to identify the minimum common bounding box that encompasses all digit morphologies.

(2) Input Encoding Process: The preprocessed 20 x 20 binary images are converted into hardware‐compatible temporal signals to drive the physical reservoir. The image is spatially segmented into 10 x 10 non‐overlapping sections, where each section consists of a 2 x 2 pixel patch. This configuration compresses the original 400‐pixel input into 100 independent reservoir nodes, which significantly reduces the input dimensionality of the reservoir computing system. In this work, the collective behavior of the 100 reservoir nodes is emulated by characterizing the dynamic current response of a representative 2T device. The encoding mechanism utilizes a 4‐bit temporal pulse train to represent the four binary pixels within each 2 x 2 patch, following a spatial z‐order. Spatially, each pixel is mapped to a specific timeframe within the pulse train, where a bit ‘1’ denotes the application of an excitatory gate pulse and ‘0’ denotes the absence of the pulse. By sequentially applying these 4‐bit pulse sequences (ranging from 0000 to 1111), the inherent volatility and short‐term memory dynamics are triggered. This process effectively transforms the spatial information of each patch into a unique read current state, which is determined by the temporal interaction of the pulse history within the device.

The resulting current responses, which serve as the high‐dimensional feature states of the reservoir, are subsequently normalized. The raw current values are scaled to [0, 1] based on the experimentally measured minimum (I_min_) and maximum (I_max_) current levels. This normalization ensures that the dynamic reservoir states are standardized for the hardware‐aware training process while preserving non‐linear differentiation produced by the temporal pulse‐device interaction.

(3) Hardware‐Aware Readout Network and Inference: The readout network is implemented as a single fully connected layer network that maps the 100‐dimensional reservoir states into 10 output digit classes. Following the training phase, the physical characteristics of the 2T devices were integrally incorporated into the readout network and inference process.

To address the representational constraints of physical devices—specifically the inability of individual transistors to express negative values—a differential weight mapping scheme was introduced. All synaptic weights were represented using the differential current of two 2T0C devices, allowing for the implementation of both positive and negative weights within the analog domain:

(3) $$\begin{array}{c} W_{ij}=G_{ij}^{1}-G_{ij}^{2} \end{array}$$

where G_ij_^1^ and G_ij_^2^ represent the conductances of the device pair.

The network is optimized by minimizing the Cross‐Entropy Loss(L):

(4) $$\begin{array}{c} L=-\Sigma T_{k}\log\left(P_{k}\right),P_{k}=\frac{e^{y_{k}}}{\Sigma e^{y_{m}}} \end{array}$$

where T_k_ represents the ground‐truth label for the k‐th digit class, and P_k_ is the predicted probability obtained via the softmax function. The term y_k_ corresponds to the logit output of the k‐th neuron, which physically represents the accumulated sum of currents from the differential device pairs in the readout array.

The training process utilizes stochastic gradient descent with a learning rate of 0.001 and a batch size of 64. The weights are updated according to the following rule:

(5) $$\begin{array}{c} W_{t+1}=W_{t}-\eta \frac{\partial L}{\partial W_{t}} \end{array}$$

where η denotes the learning rate. Throughout the optimization, the update trajectory is managed to ensure the resulting weight states remain strictly within the physical operational window of the device. This hardware‐constrained learning approach prevents the model from converging to states that are not physically achievable, thereby ensuring high reliability during actual hardware deployment.

### 4.5 Motion Simulation Methods

(1) Motion Dataset Composition and Preprocessing: To evaluate the spatiotemporal recognition capabilities of the proposed system, a motion dataset was constructed from four directional trajectories—Up, Down, Left, and Right—across six distinct spatial positions arranged along the rows or columns, resulting in 24 motion scenarios. For each motion scenario, 100 repeated current responses were experimentally measured, yielding a total of 2400 measured response traces. Before data augmentation, the 100 measured responses for each scenario were randomly partitioned into training, validation, and test subsets containing 64, 16, and 20 responses, respectively. Data augmentation was applied exclusively to the training subset. For each scenario, the mean reservoir‐state vector and covariance matrix were estimated from the 64 measured training responses, and synthetic state vectors were sampled from the corresponding multivariate Gaussian distribution. The magnitudes of the sampled state values were used to construct 640 training samples per scenario, yielding 15 360 training samples in total. The same current‐magnitude representation was applied to the experimentally measured validation and test responses, which were used without augmentation, yielding 384 validation and 480 test samples, respectively. Each motion event was represented by a time‐varying current signal corresponding to the transient interaction between the stimulus and the physical reservoir. Virtual nodes were uniformly extracted from the current traces across the six‐pulse response window, with each node representing a distinct temporal state of the physical reservoir. Normalization parameters were determined exclusively from the augmented training data and subsequently applied to the validation and test data.

(2) Network Training and Simulations: Direction and position classification was performed using two independent readouts connected to the virtual reservoir states, with four output classes for direction and six output classes for position. For the principal RC analysis, ridge regression‐based linear readouts were used with a fixed regularization parameter of α = 1.0. The reservoir dimension was selected based on the joint direction‐and‐position accuracy on the validation set, resulting in *n* = 27 for the final RC configuration. The corresponding ridge readouts directly mapped the reservoir states to the direction and position outputs without a trainable hidden layer. Softmax regression and MLP readouts were additionally evaluated as complementary comparisons of readout complexity. For these comparisons, the networks were trained by minimizing the cross‐entropy loss using the Adam optimizer with a learning rate of 0.01 and full‐batch training, targeting a joint direction‐and‐position validation accuracy of 95%.

For hardware‐aware readout evaluation, each signed readout weight was mapped onto a differential conductance pair, with the effective weight proportional to the conductance difference between two devices, *G* ^+^ − *G* ^−^. Both *G* ^+^ and *G* ^−^ were selected from discrete conductance states obtained from the experimentally measured potentiation characteristics of the 2T memory. The continuous readout weights were normalized and assigned to the nearest available differential‐conductance level. The resulting hardware‐mapped weights were used to evaluate the VMM read energy of the readout array. Detailed procedures for data partitioning and augmentation, ridge regression evaluation, differential‐conductance mapping, and VMM read‐energy calculation are provided in Note S3.

## Author Contributions

**Jeong‐Min Park**: conceptualization, methodology, data curation, validation, investigation, formal analysis, writing – original draft, writing – review and editing. **Yeong‐In Kim**: methodology, software, data curation, investigation, validation, formal analysis, writing – review and editing, writing – original draft. **Jeong Hyun Yoon**: formal analysis, validation. **Sein Lee**: validation, formal analysis. **Wooho Ham**: methodology, validation. **Dongyun Lee**: investigation. **Junseo Lee**: validation. **Dongwook Shin**: validation. **Kyung Jun Park**: formal analysis. **Seok Daniel Namgung**: validation, writing – review and editing, formal analysis, supervision. **Min‐Kyu Song**: writing – review and editing, visualization, supervision. **Jang‐Yeon Kwon**: writing – review and editing, project administration, supervision, funding acquisition, resources.

## Funding

This work was supported by BK21 FOUR (Fostering Outstanding Universities for Research) funded by the Ministry of Education (MOE) of Korea. This work was supported by the National Research Foundation of Korea (NRF) grant funded by the Korea government (MSIT) (Grant No. RS‐2024‐00351713).

## Conflicts of Interest

The authors declare no conflicts of interest.

## Supporting information




**Supporting File 1**: advs77921‐sup‐0001‐SuppMat.docx.

**Supporting File 2**: advs77921‐sup‐0002‐VideoS1.mp4.

## Data Availability

The data that support the findings of this study are available from the corresponding author upon reasonable request.
