# Supplementary material for: Ultralow‐Energy Analog Reservoir Computing via Reconfigurable 2‐Transistor Memory
Source: Adv Sci (Weinh). 2026 Sep 29:e77921. Online ahead of print. doi: 10.1002/advs.77921 (PMC13621255; doi:10.1002/advs.77921)
Supplement: Supplementary file 1 [file ADVS-9999-e77921-s001.docx]

Supporting Information

**Ultralow-Energy Analog Reservoir Computing via Reconfigurable 2-Transistor Memory**

Jeong-Min Park^1,2,†^, Yeong-In Kim^3,†^, Jeong Hyun Yoon^4^, Sein Lee^4^, Wooho Ham^1,2^, Dongyun Lee^1,2^, Junseo Lee^1,2^, Dongwook Shin^1,2^, Kyung Jun Park^1,2^, Seok Daniel Namgung^5^, Min-Kyu Song^3^* and Jang-Yeon Kwon^1,2^*

1 School of Integrated Technology, Yonsei University, Incheon, 21983 Republic of Korea

2 BK21 Graduate Program in Intelligent Semiconductor Technology, Yonsei University, Incheon, 21983 Republic of Korea

3 School of Electrical Engineering, Korea University, 145 Anam-ro, Seongbuk-gu, Seoul, 02841 Republic of Korea

4 Department of Materials Science and Engineering, Seoul National University, Seoul, 08826 Republic of Korea

5 School of Electrical and Electronics Engineering, Chung-Ang University, Seoul, 06974 Republic of Korea

† These authors contributed equally to this work.

* To whom correspondence should be addressed:

Prof. Min-Kyu Song, Ph.D.

School of Electrical Engineering, Korea University, 145 Anam-ro, Seongbuk-gu, Seoul 02841, Republic of Korea

E-mail: [mksong@korea.ac.kr](mailto:mksong@korea.ac.kr)

Prof. Jang-Yeon Kwon, Ph.D.

School of Integrated Technology, Yonsei University, Incheon 21983, Republic of Korea

E-mail: [jangyeon@yonsei.ac.kr](mailto:jangyeon@yonsei.ac.kr)

**Table S1** Comparison with other state-of-the-art results in reservoir systems

**Figure S1.** Comparison with other vector-matrix multiplication (VMM) cores.^[10]^

**Figure S2.** Gate-bias-stress stability and gate leakage characteristics of the ITZO TFT. a) Transfer characteristics measured before and during positive bias stress at V_G_ = +15 V for 1 h. The transfer curves were recorded at 0, 600, 1800, and 3600 s with V_D_ = 0.1 V, yielding ΔV_1nA_ = 0.34 V after 1 h. b) Transfer characteristics measured before and during negative bias stress at V_G_ = −15 V for 1 h. The transfer curves were recorded at 0, 600, 1800, and 3600 s with V_D_ = 0.1 V, yielding ΔV_1nA_ = 0.24 V after 1 h. c) Gate leakage currents measured during the positive bias-stress test. d) Gate leakage currents measured during the negative bias-stress test. Under both stress conditions, the gate leakage current remained on the order of 10^-12^-10^-11^ A, close to the instrumental detection limit of approximately 10^-11^ A, without a systematic increase or abrupt rise associated with dielectric breakdown.

**Figure S3.** Systematic V_TH_ tuning via oxygen partial pressure control during RF sputtering. Positive bias stress stability of TFT in PO_2_ condition of a) 3.32 % and b) 1.66 %.

**Figure S4.** Raw readout-current traces for the programmed ‘1’ and ‘0’ states used in retention evaluation. Current responses prior to conversion into the storage-node voltage (V_SN_) for the retention measurements shown in Figure 2d.

**Figure S5.** a) Experimental data and fitted line used for Current-to-V_SN_ conversion. b) Current-to-V_SN_ conversion equation used for retention analysis.

**Note S1** Current-to-V_SN_ conversion

To quantify retention in the voltage domain, the readout current was converted to the storage-node voltage (V_SN_) using an experimentally derived calibration based on the transfer characteristics of the read transistor. As shown in Figure S5a, we used the measured transfer curve (I_D_-V_G_, black) obtained at V_D_ = 0.1 V while sweeping V_G_ from 1 to 5 V. Because the read operation satisfies the linear-region condition $\left( V_{DS}<V_{G}-V_{TH} \right)$, the drain current is governed by the linear-regime relation:

$$\begin{aligned} I_{D}=\mu C_{i}\frac{W}{L}\left( V_{G}-V_{TH} \right)V_{D}\#(1) \end{aligned}$$

where $\mu$ is the field-effect mobility, $C_{i}$ is the gate-insulator capacitance per unit area, $W/L$ is the channel aspect ratio, and $V_{D}$ corresponds to the applied drain bias during readout.

Accordingly, the experimentally measured transfer data in the linear regime were fitted with a linear function (red line in Figure S5a), yielding a monotonic mapping between $I_{D}$ and V_SN_. The extracted fitting equation is summarized in Figure S5b.

During retention measurements, the sampled read currents for the programmed ‘1’ state (Figure S4) were converted into the corresponding V_SN_ values by inverting the fitted linear relation. All current data were measured under V_RBL_ = 0.1 V to match the reading voltage. The resulting V_SN_(t) trajectories were then used to evaluate retention and to define the retention time as the time which V_SN_ decreased to 90% of its initial value.^[11]^

**Figure S6.** Retention characteristics of the programmed ‘0’ state as a function of V_hold_. Complementary data to Figure 2e, showing how the ‘0’ state transitions from quasi-non-volatile to volatile behavior as V_hold_ approaches and exceeds 0 V.

**Figure S7.** Device-to-device distributions of a) subthreshold swing, b) field-effect mobility, and c) V_TH_ measured across 20 devices from a single fabrication batch (n = 20 devices).

**Figure S8.** Current-domain representation of multi-level quasi-non-volatile retention. Raw current data corresponding to the 3-bit retention states in Figure 2f, programmed by sweeping WBL from 1.0 to 2.4 V (0.2 V step).

**Figure S9.** (a) Write-transistor current measured under an externally applied drain bias as a function of gate voltage. Because this measurement does not reproduce the floating-node boundary condition during retention, it is used only to indicate the qualitative leakage/conduction tendency with V_hold_ and not as the absolute storage-node relaxation current, I_relax_. (b) Corresponding change in storage-node voltage, ΔV_SN_ = V_SN_(t) − V_SN,0_, obtained using the experimentally calibrated current-to-V_SN_ relationship, directly showing the V_hold_-dependent relaxation of the storage node.

**Figure S10.** Detailed operating waveforms for optimized volatile operation. Voltage waveforms applied to a) WBL and b) WWL for the optimized potentiation/relaxation pulse scheme used in Figure 3g–3h. 30 voltage-driven potentiation pulses and 30 leakage-driven pulses (no bias except 0.1 V read at RBL) are shown.

**Figure S11.** Definition of evaluation points for 4-bit physical-reservoir states. Illustration of the four sampling points (blue markers) used to extract the output state at the fourth pulse in Figure 4a.

**Figure S12.** Example input voltage waveform for a representative 4-bit pattern. Applied voltage waveform at a) WBL and WWL, and b) RBL and RWL for the 1110 input state.

**Figure S13.** Full dataset of cycle-to-cycle variation for 4-bit reservoir outputs. Measured current distributions for all 16 states (0000–1111) repeated 20 times on the same device, supporting the statistical analysis in Figure 4b–4c.

**Figure S14.** Full dataset of device-to-device variation for 4-bit reservoir outputs. Measured current distributions a) 1100, b) 1101, c) 1110, d) 1111 states collected from 12 devices from a single fabrication batch, supporting Figure 4e–4f.

**Figure S15.** a) Confusion matrix and b) training loss for MNIST handwritten-digit recognition. Loss evolution versus training epochs for the reservoir-computing evaluation described in Figure 5c–5d.

**Note S2** Energy Consumption Calculation: MNIST

The read energy consumption of the single volatile 2T physical reservoir for the handwritten digit recognition task is quantitatively estimated as follows:

$$\begin{aligned} E_{reservoir} = V_{read}\times I_{2T,reservoir} \times t_{width} \end{aligned}$$

$$\begin{aligned} = 0.1 V \times477.02 nA \times1 \mu s = 47.7\frac{fJ}{pulse} \end{aligned}(2)$$

where V_read_ is the read voltage applied during reservoir-state sensing, I_2T,res_ is the average current response of the volatile 2T reservoir device under the 4-bit temporal input sequence, and t_width_ is the pulse width. Here, we use I_2T,res_ = 477.02 nA, estimated from the current evolution characteristics in Figure 4a (ranging from ~168 nA to ~779 nA). Such read energy consumption is significantly lower than those of state-of-the-art physical reservoirs used for hardware-based analog reservoir computing (Table S1).

The VMM read energy of a single quasi-non-volatile 2T readout cell is quantitatively estimated as follows:

$$\begin{aligned} E_{readout} = {V_{read}}^{2}\times G_{2T,readout} \times t_{width} \\ = \left( 0.1 V \right)^{2}\times661.07 nS \times1.5 \mu s = 9.92\frac{\mathrm{fJ}}{\mathrm{pulse}}\#(3) \\ \end{aligned}$$

where G_2T,NV_ is the average conductance of the quasi-non-volatile 2T readout cell used for vector–matrix multiplication. This form follows I = G × V under small-bias readout, leading to P = V×I = V^2^×G. We assume G_2T,NV_ = 661.07 nS based on the experimentally measured conductance modulation characteristics (Figure 2g) of the quasi-non-volatile 2T device (ranging from ~36 nS to ~855 nS).^[3]^

**Figure S16.** Multi-V_hold_ kernel voltage assignment. Numbers inside each box indicate the applied V_hold_ value (in volts), supporting the concept shown in Figure 6b.

**Figure S17.** Joint 24-class confusion matrix for motion classification using the physical reservoir with a ridge readout (N = 27). The 24 joint motion classes comprise four motion directions (Right, Left, Down, and Up) across six spatial positions. A prediction was considered correct only when both the motion direction and spatial position were correctly identified.

**Figure S18**. Comparison of readout architectures and computational requirements for the physical reservoir. a) Training epochs required for the softmax-regression readout to reach the target joint direction-and-position validation accuracy as a function of the number of reservoir nodes. The required training epochs gradually decreased with increasing reservoir dimension. b) Training epochs required for single-hidden-layer MLP readouts with varying reservoir dimension (N) and hidden-layer size (H). The required training epochs depended on both reservoir dimensionality and readout-network size. c) Comparison of the VMM read energy for the selected softmax-regression (N = 24), ridge-regression (N = 27), and single-hidden-layer MLP (N = 4, H = 16) configurations. d) Comparison of the training epochs required for the selected readout configurations. Ridge regression uses a closed-form solution without iterative training, whereas the softmax-regression and MLP readouts require iterative optimization.

**Figure S19.** Comparison of VMM read energy for non-reservoir input representations across different readout architectures. a) VMM read energy for the 216-dimensional direct spatial-input representation using ridge-regression, softmax-regression, and single-hidden-layer MLP readouts with varying hidden-layer size (H). b) VMM read energy for the 180-dimensional consecutive frame-difference representation using the same readout architectures. The VMM read-energy requirement varies with both the input representation and readout-network size.

**Figure S20.** Estimated reservoir read energy during the motion-detection operation.

**Note S3** Computational Analysis of the Motion Task

1. Dataset Construction and Data Augmentation

The proposed reservoir computing (RC) framework was evaluated using a spatiotemporal motion dataset comprising 24 motion scenarios defined by four motion directions and six spatial positions. For each scenario, the device response was experimentally measured over 100 repeated runs, yielding a total of 2,400 measured response traces. For each randomized data partition, the 100 measured runs per scenario were divided into 64 training, 16 validation, and 20 test runs before data augmentation, with the same run-index split applied across all 24 motion scenarios. Data augmentation was then performed on the training subset by estimating the mean reservoir-state vector and covariance matrix from the 64 measured training runs for each scenario and generating 640 synthetic state vectors through multivariate Gaussian sampling. The magnitudes of the resulting state values were used for classification, while the validation and test subsets were constructed from the measured responses without augmentation. Normalization parameters were determined from the augmented training data and subsequently applied to the validation and test data.

This partitioning and augmentation procedure was repeated across 100 randomized data partitions using random seeds from 0 to 99, with the same measured-run partition maintained across all compared reservoir configurations within each repetition. Classification performance was subsequently evaluated across the 100 randomized partitions and reported as the mean and standard deviation. The 95 % confidence interval was calculated based on the 100 randomized partitions and the measured training-to-test split (64:20). For the selected N = 27 reservoir configuration, the joint direction-and-position test accuracy of 98.09 ± 1.78 % corresponded to a 95 % confidence interval of 96.10–100.00 %.

2. Readout Training and Energy Analysis

The reservoir states were classified using separate ridge-regression-based linear readouts for motion direction and spatial position. The respective readouts mapped the N-dimensional reservoir state vector to four- and six-dimensional outputs, with the predicted class determined from the maximum readout value. A fixed ridge regularization strength of α=1.0 was used for the RC readouts. For the additional softmax regression and MLP readout comparisons, cross-entropy loss and the Adam optimizer with a learning rate of 0.01 were used with full-batch training. Training was terminated when the joint direction-and-position validation accuracy reached the target of 95 % or when no further improvement was observed for 500 consecutive epochs. For comparison with the reservoir representation, a direct spatial-input representation was constructed by flattening the six 6 × 6 binary motion frames into a 216-dimensional vector without reservoir encoding. A frame-difference representation was constructed from the pixel-wise differences between consecutive frames, resulting in a 180-dimensional input vector.

After the ridge-regression weights were obtained, the continuous weights were mapped onto the experimentally measured conductance states of the 2T memory using a differential representation, $W\propto G^{+}-G^{-}$. The available differential-conductance levels were determined from all possible combinations of $G^{+}$ and $G^{-}$, each selected from the 30 discrete conductance states obtained from the measured potentiation characteristics. For combinations yielding the same effective differential weight, the pair with the lowest total conductance, $G^{+}+G^{-}$, was retained. The resulting differential-conductance levels were normalized to the range from −1 to 1, while the weights of each readout were independently normalized by their maximum absolute value and assigned to the nearest available differential-conductance level.

The VMM read energy of the hardware-mapped readout array was calculated from the conductance states assigned to the differential weights as

$$E_{\mathrm{VMM}}=V_{\mathrm{read}}^{2}t_{\mathrm{read}}\sum_{i,j} (\left. G_{ij}^{+}+G_{ij}^{-} \right).$$

All input rows were simultaneously activated during the calculation, using the mapped $G^{+}$ and $G^{-}$ conductance states for each differential weight. The VMM read energy was evaluated across the 100 randomized data partitions used for the ridge classification analysis and reported as the mean and standard deviation.

The computational codes for data preprocessing, ridge-regression-based classification, hardware-aware weight mapping, and energy analysis are available through a public GitHub repository.

https://github.com/IYK02608/MotionClassification

**Note S4 Trainable-Weight Comparison in the Motion-Detection Task**

To provide a transparent comparison, we define the trainable-weight count used in Figure 6. The comparison is limited to the trainable readout arrays and excludes peripheral circuitry.

1. Trainable-weight count

The RC readout uses 27 reservoir-derived features:

- Position: 27 × 6 = 162
- Direction: 27 × 4 = 108
- Total RC weights = 270

The ANN directly uses the 216-dimensional input:

- Position: 216 × 6 = 1,296
- Direction: 216 × 4 = 864
- Total ANN weights = 2,160

Therefore, 2,160 / 270 = 8.00, indicating an 8.00-fold reduction in trainable weights.

2. Device-array-level read energy

- RC: 4.26 pJ
- ANN: 16.95 pJ

Thus, 16.95 / 4.26 = 3.98, corresponding to a 3.98-fold reduction in device-array-level read energy.

**Table S2. Trainable-weight and energy comparison**

| Accounting level | RC | ANN | ANN/RC |
| --- | --- | --- | --- |
| Input features | 27 | 216 | 8.00× |
| Position weights | 162 | 1,296 | 8.00× |
| Direction weights | 108 | 864 | 8.00× |
| Total trainable weights | 270 | 2,160 | 8.00× |
| Device-array-level read energy | 4.26 pJ | 16.95 pJ | 3.98× |

3. Non-trainable hardware and peripheral circuits

The trainable-device comparison does not represent the complete hardware required to operate either architecture. The RC architecture additionally requires a physical reservoir, V_hold_ generation, input routing, temporal control, reservoir-state sampling, and associated interface circuitry. Likewise, a complete ANN implementation requires input-routing circuits, neuron and activation circuits, array drivers, sensing circuits, data converters, control logic, and other peripheral components.

Because these non-trainable and peripheral circuit blocks were not physically implemented or experimentally characterized in the present work, their transistor counts, areas, and energy consumption cannot be reliably quantified without introducing additional circuit-level assumptions.

Therefore, the reported 8.00-fold reduction is restricted to the trainable synaptic weights under identical implementation assumptions.

4. Energy-accounting boundary

The reported energy comparison includes only the device-array read energy of the trainable readout networks.

Under this identical device-array accounting boundary, the estimated read energies are: RC readout: 4.26 pJ, ANN baseline: 16.95 pJ, corresponding to 16.95 pJ / 4.26 pJ = 3.98.

Thus, the proposed RC architecture provides a 3.98-fold reduction in estimated device-array-level read energy relative to the ANN baseline under the defined accounting conditions.

**Figure S21.** Self-aligned top-gate (SATG) ITZO TFT architecture for the 2T platform.

**Figure S22.** Fabrication flow of the ITZO SATG TFT. Process sequence summarizing ITZO deposition/patterning/anneal, gate-stack formation, self-aligned etching, Ar-plasma channel metallization, and W source/drain formation.

**Figure S23.** a) Top-view of SATG structure utilized for 2T cell. Cross-sectional b) TEM and c) EDS profile of the SATG stack. TEM side-view image with corresponding EDS line profile across channel/gate insulator/gate regions, confirming the layered structure and interfaces.

**Figure S24.** Global tight-cropping procedure for MNIST preprocessing. Illustration of dataset-wide bounding-box extraction and cropping that converts original 28 × 28 digits into 20 × 20 binary images for hardware-compatible encoding.

**References**

[1]	W. Ahn, S. Lee, J. Oh, H. Lee, S. Choi, *Adv. Mater.* **2025**, 37.

[2]	Z. Chen, W. Li, Z. Fan, S. Dong, Y. Chen, M. Qin, M. Zeng, X. Lu, G. Zhou, X. Gao, *Nat. Commun.* **2023**, 14, 3585.

[3]	J. Kim, E. C. Park, W. Shin, R.-H. Koo, C.-H. Han, H. Y. Kang, T. G. Yang, Y. Goh, K. Lee, D. Ha, S. S. Cheema, J. K. Jeong, D. Kwon, *Nat. Commun.* **2024**, *15*, DOI 10.1038/s41467-024-53321-2.

[4]	S. Zhang, J. Zhu, R. Qiu, D. Liu, Q. Ren, M. Zhang, *Adv. Mater.* **2025**, *37*, DOI 10.1002/adma.202418418.

[5]	D. Kim, S. Lee, Y. Lee, Y. Park, J. Lee, S. Kim, *Adv. Funct. Mater.* **2024**, *34*, DOI 10.1002/adfm.202409095.

[6]	S. Kim, D. H. Shin, W. Choi, S. Cheong, S. K. Shim, S. H. Lee, J. Han, Y. H. Jang, K. Son, N. Ghenzi, C. S. Hwang, *Adv. Mater.* **2026**, *38*, DOI 10.1002/adma.202510635.

[7]	R. Koo, C. Han, J. Yim, J. Im, Y. Cho, J. Lee, S. S. Cheema, J. Kim, W. Shin, D. Kwon, *Adv. Mater.* **2026**, *38*, DOI 10.1002/adma.202511337.

[8]	A. Chowdhury, A. Rasyotra, H. Ravichandran, D. K. Manoharan, Y. Sun, Y. Yang, S. Das, Nat. Commun. **2025**, 16, 10252.

[9]	K. Liu, T. Zhang, B. Dang, L. Bao, L. Xu, C. Cheng, Z. Yang, R. Huang, Y. Yang, Nat. Electron. **2022**, 5, 761.

[10]	F. Aguirre, A. Sebastian, M. Le Gallo, W. Song, T. Wang, J. J. Yang, W. Lu, M.-F. Chang, D. Ielmini, Y. Yang, A. Mehonic, A. Kenyon, M. A. Villena, J. B. Roldán, Y. Wu, H.-H. Hsu, N. Raghavan, J. Suñé, E. Miranda, A. Eltawil, G. Setti, K. Smagulova, K. N. Salama, O. Krestinskaya, X. Yan, K.-W. Ang, S. Jain, S. Li, O. Alharbi, S. Pazos, M. Lanza, *Nat. Commun.* **2024**, *15*, DOI 10.1038/s41467-024-45670-9.

[11]	Q. Hu, C. Gu, Q. Li, S. Zhu, S. Liu, Y. Li, L. Zhang, R. Huang, Y. Wu, *Adv. Mater.* **2023**, *35*.
